# Local dynamical survival analysis and human sensor networks for epidemic tracking and forecasting

**DOI:** 10.1098/rsif.2026.0111

**Published:** 2026-10-28

**Authors:** Micaela Richter, Prescott Alexander, Grzegorz Rempala, Eben Kenah

**Affiliations:** ^1^ Division of Biostatistics, College of Public Health, The Ohio State University, Columbus, OH, USA; ^2^ Information Systems and Modeling, Los Alamos National Laboratory, Los Alamos, NM, USA

**Keywords:** epidemics, epidemiology, forecasting, surveillance, survival analysis, human sensor network

## Abstract

Predictive modelling for infectious diseases remains an important problem in epidemiology. Current approaches are often limited by their dependence on time-series data, which can lead to biased estimates of key epidemiological parameters. Here, we present an extension of the dynamical survival analysis approach that can accommodate epidemic data from a large-scale, complex, agent-based model. We use localized predictions that allow for flexibility with time-dependent parameters to account for human behaviour and environmental factors during the epidemic. Our results demonstrate that it is possible to evaluate the quality of predictions and highlight the potential benefits of surveillance via longitudinal follow-up of individuals sampled from the population, which we call a human sensor network.

## 1. Introduction

In an era of climate volatility, political instability and global mobility, the risk from emerging and re-emerging infectious diseases with wide geographic spread and rapid growth continues to increase [1,2]. The recent COVID-19 pandemic illustrated the extent to which a novel pathogen can rapidly overwhelm healthcare systems and cause millions of deaths worldwide. Currently, the re-emergence of measles in the USA and Canada has underscored the ongoing challenges of disease surveillance and control [3,4].

Forecasting is a central focus of epidemic modelling and remains a significant challenge owing to factors such as heterogeneity in the population and changing human behaviour [5]. Various approaches have been developed to analyse epidemic data and to model disease dynamics while accounting for such factors. During the COVID-19 pandemic, statistical analyses using regression models based on time-series data were most common [6]. Other modelling studies fitted compartmental models to time-series data [7,8] or used stochastic transmission models [9,10]. Beyond this, network-based models and agent-based models [11,12] were developed to better address various data types and research goals.

Regardless of the model choice, the quality of the data collected during an epidemic is crucial in determining model efficacy. Available data during an epidemic usually consist of reported case counts, death counts and contact tracing information, which can inform assumptions about infection periods and latent periods. Typically, these data are susceptible to several types of bias, many of which are related to incompleteness and limited knowledge about the disease. When observations occur in the beginning of an outbreak, these biases can be especially large [13]. Delays in reporting, selective reporting and underreporting can all result in biased estimates of key parameters used in epidemic modelling [14], such as the basic reproduction number ($R_{0}$).

There are also less obvious sources of bias that can arise when collecting and interpreting time-series epidemic data. For example, looking backward in time for contact tracing can bias estimation of the generation interval, which is the time between successive infections (i.e. the infection time of an infector and the infection time of an infectee). Generation intervals are commonly used to estimate $R_{0}$ and effective reproduction numbers ($R_{t}$). At the start of an epidemic, cases are growing exponentially, so using contact tracing to estimate generation intervals will result in an over-representation of short generation intervals and an under-representation of long generation intervals [14]. Furthermore, the generation interval is not an observable quantity in practice, so the serial interval—the time between successive symptom onsets—is used as a proxy instead. However, this substitution can bias epidemiological estimates as these two quantities have important differences. Serial and generation intervals often have the same means but different variances [14,15]. Serial intervals can be negative in some cases, whereas generation intervals must always be positive. Although there have been various methods proposed to correct these biases [14–16], obtaining reliable insights that can be used to inform policy remains a challenge. Despite notable advances, more work is needed to implement systematic and accurate epidemic data tracking systems and strategies for inference.

Here, we present an extension of dynamical survival analysis (DSA) [17–19] and demonstrate its application to a random sample of the population followed longitudinally, which we call a human sensor network. The times at which each participant transitions between disease states (susceptible, exposed, infected and recovered) are recorded, and these data provide a rapid and representative picture of how the epidemic is spreading in the population. In the DSA approach, these data are used to construct a likelihood function to estimate the parameters (e.g. rates of infection and recovery) of a population-level model of epidemic spread [17]. We extend the original DSA framework by allowing these parameters to change over time and estimating them using DSA likelihoods based on data within a sliding time window. Under this approach, the behaviour of a complex epidemic model can be approximated by a much simpler and more numerically stable model with time-varying parameters.

We first demonstrate the reliability of this approach, which we call local DSA, when fitted to data generated from a compartmental susceptible–exposed–infected–recovered (SEIR) epidemic model. We then demonstrate its performance when fitted to data generated from a complex agent-based model that does not have a compartmental SEIR structure, reflecting the uncertainty about population-level dynamics often present in a real epidemic. A flexible approach like local DSA can yield stable estimates of transmission dynamics and provide insight into the quality of its own predictions, even under challenging conditions. This work introduces two complementary tools for epidemiological analysis: human sensor networks and local DSA. Together, these hold tremendous promise for guiding the public health response to future epidemics.

## 2. Methods

### 2.1. Overview

To explore the performance of local DSA under favourable circumstances, we implemented it using infection trajectories generated from a compartmental SEIR model. To explore the performance of local DSA under more realistic conditions, we implemented it using data generated from a large-scale, agent-based model of respiratory disease spread in the USA created at Los Alamos National Laboratory [20]. This model, Epicast, is designed to replicate real-world conditions and thus the data, although still from a simulation, are meant to represent a much more realistic outcome of an epidemic. We evaluated the model’s performance based on parameter estimation and prediction ability.

### 2.2. Model

In all cases, the DSA likelihood was based on a compartmental SEIR model where the flow of individuals in the population between the compartments S, E, I and R is determined by the following set of ordinary differential equations (ODEs):

(2.1) $$\begin{array}{rl} & \dot{S_{t}}=-\beta S_{t}I_{t}\text{,}\\ & \dot{E_{t}}=\beta S_{t}I_{t}-\delta E_{t}\text{,}\\ & \dot{I_{t}}=\delta E_{t}-\gamma I_{t}\;\text{and}\\ & \dot{R_{t}}=\gamma I_{t} \end{array}$$

with initial conditions ($S_{0}=1-\rho ,\;E_{0}=\rho_{E},\;I_{0}=\rho_{I},\;R_{0}=\rho_{R},\;\rho =\rho_{E}+\rho_{I}+\rho_{R}$). The parameters are the infectious contact rate β, rate of infectiousness onset δ, the recovery rate γ and the initial conditions ($\rho_{E}$, $\rho_{I}$ and $\rho_{R}$).

In the DSA approach, solutions of these differential equations can be used to estimate model parameters using a likelihood based on data from a human sensor network and tools from survival analysis [17,19]. In the DSA framework, we reinterpret equation (2.1) from an individual-level rather than a population-level point of view. For example, $S_{t}$ no longer represents the proportion of the population that remains susceptible at time t. Instead, it represents the survival function for a randomly selected susceptible individual in the population at time t. Similarly, $R_{0}r_{t}=\beta \int_{0}^{t}\iota_{u}\;du$ (where $\int_{0}^{t}\iota_{u}\;\text{d}u$ is the deterministic limit of $\int_{0}^{t}I(u)/n\;\text{d}u$) can be interpreted as the cumulative hazard of infection. We can derive density functions for each event in an individual’s disease trajectory (exposure, onset of infectiousness and recovery) and use these to construct a likelihood. A more detailed derivation of the likelihood function for DSA is described in §2.6. The DSA framework allows us to incorporate individual heterogeneities into the model that would otherwise have been aggregated out and to accurately quantify uncertainty in parameter estimates and predictions.

### 2.3. Synthetic data

We generated synthetic data by simulating infection trajectories for N individuals from an SEIR model. First, we simulated an SEIR epidemic by numerically solving the Kermack–McKendrick ODEs (equation (2.1)), given a set of values for the parameters β, δ, γ and initial conditions. We chose parameter values and initial conditions to be representative of a typical respiratory pathogen: a reproduction number $R_{0}=\beta /\gamma$ of 2.5, a mean latent period of ≈3 days and a mean infectious period of 5 days [21,22]. Unless otherwise specified, all simulations using synthetic data used the parameter values $\beta =0.5,\delta =0.3,\gamma =0.2,\rho_{E}=0.02,\rho_{I}=0.01\text{ and }\rho_{R}=0.02$.

Next, we began creating the human sensor network by selecting an observation window $\left(t_{\text{start}},\;t_{\text{stop}}\right]$ and a random sample of individuals. For each individual, exposure times were generated using the inverse cumulative distribution function method. Uniform random variables on [0,1] were mapped to exposure times via the inverse of the cumulative incidence curve, 1−S(t). Then, latent periods were drawn from an exponential distribution with rate δ and infectious periods were drawn from an exponential distribution with rate γ. Adding latent periods to exposure times yields an infectiousness onset time, and adding the infectious period to the infectiousness onset time yields the recovery time. If an event did not occur for an individual or if it happened outside of the observation window (i.e. left- or right-censored), then the event time was set to the minimum or maximum window time and the event indicators were set as explained below. Thus, each individual had follow-up times $\left\{t_{E},t_{I},t_{R}\right\}$ and event indicators $\left\{1_{E},1_{I},1_{R}\right\}$. Unless otherwise specified, we used 5000 human sensors (i.e. participants in the human sensor network) for each simulation.

### 2.4. Epicast data

A second dataset was used from a simulation called Epicast. Epicast is a large-scale, agent-based model of respiratory disease spread created at Los Alamos National Laboratory. It uses a highly detailed, synthetic population of the USA to simulate the movements and interactions of over 320 million agents across 50 states [20,23]. Epicast replicates the contact patterns of individuals in the USA as they interact with each other in workplaces, homes, schools and community settings to produce simulated outbreaks across a wide range of scenarios, allowing for user specification of policy interventions and parameter tuning. The model provides insights into various epidemic scenarios with high granularity and demographic heterogeneity. Notably, the high complexity of Epicast is in sharp contrast to the synthetic data described above. For more information on Epicast, see [20].

We tested local DSA on a human sensor network sampled from an Epicast run that simulated the entire state of Ohio, containing approximately 11 million agents. For simplicity and initial testing, we did not include any vaccination or policy intervention scenarios, although the population and spatial heterogeneity alone added some realism to the epidemic. Using the Epicast data to test our combination of human sensor networks and local DSA provided a more rigourous evaluation of the method’s utility in a real epidemic.

### 2.5. Human sensor networks

The DSA model framework uses individual histories of exposure, infectiousness onset and recovery times. These are obtained from a human sensor network, which is a random sample of the population that is tracked and observed longitudinally. In practice, this could involve frequent home visits, a remote testing and reporting system (e.g. home testing kits and mobile reporting of symptoms, etc.) or a combination of both. This approach to epidemic surveillance avoids many challenges present in time-series data. First, a human sensor network is representative by construction, and the sampling from the population can incorporate all of the standard tools of survey sampling. Second, the data are timely since they are being collected as often as is feasible given the pathogen characteristics and logistical capacity. Third, repeated longitudinal testing allows for much better capture of mild or asymptomatic cases, giving a more accurate picture of the clinical spectrum of infection. Fourth, a human sensor network can directly monitor changes in behaviour, so it is much less susceptible to biases caused by changes in test seeking or health seeking behaviour than traditional surveillance [24].

Cohort studies approximating a human sensor network have been implemented for disease surveillance [25–27], but they have not incorporated the frequency of observation and testing that is possible in a human sensor network. Although logistically challenging, this type of surveillance has the potential to provide unmatched reliability, detail and timeliness for making policy decisions in an epidemic.

In our simulated human sensor networks, we assume that we observe each individual’s exact exposure, infectiousness onset and recovery times. In practice, we would probably only observe their status (susceptible, exposed, infectious and recovered) through testing at discrete times. We select a random sample of the entire population and an observation window denoted by $\left(t_{\text{start}},t_{\text{stop}}\right]$. During the observation window, we may observe an event time for an individual, or the event could be left- or right-censored depending on the timing of each agent’s transitions. There is a bias–variance trade-off in the width of the window: narrow windows have lower bias but higher variance whereas wide windows have higher bias but lower variance. For our simulated data, we used a window of 5 days. For the Epicast data, we chose a window of 7 days to account for weekly effects present in the population.

It is critical to handle the event indicators $\left\{1_{E},1_{I},1_{R}\right\}$ correctly to account for the left- and right-censoring of event times. There are 10 possible observation cases based on the timing of each transition relative to the observation window. Figure 1 displays each case on a timeline with observed event times marked in black, while left- or right-censored event times are in grey. Each case yields a different likelihood contribution, and the overall likelihood is the product of the contributions from all participants in the human sensor network.

**Figure 1.**
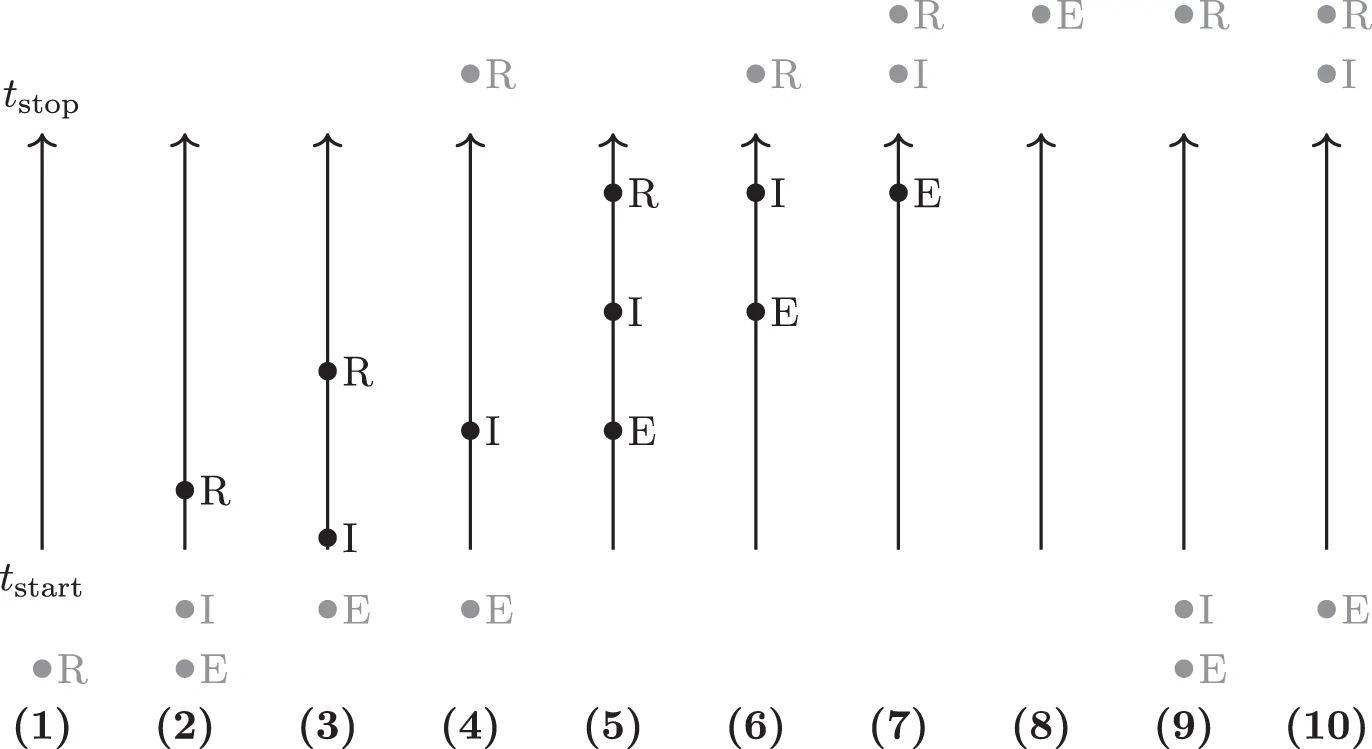
In an SEIR model, there are 10 possible ways to be observed in a window denoted by $\left(t_{\text{start}},t_{\text{stop}}\right]$. Grey points before $t_{\text{start}}$ are left-censored, and grey points after $t_{\text{stop}}$ are right-censored. Points on the line in black are observed exactly.

Let $\left\{T_{E},T_{I},T_{R}\right\}$ denote the true transition times (from S to E, from E to I and from I to R, respectively), and let $\left\{t_{E},t_{I},t_{R}\right\}$ denote the observed times recorded in the study. If a true event time $T_{X}$ occurs before $t_{\text{start}}$, then we set the observed event time $t_{X}=t_{\text{start}}$ with event indicator $1_{X}=1$. If a time $T_{X}$ occurs after $t_{\text{stop}}$, then we set the observed event time $t_{X}=t_{\text{stop}}$ with the event indicator $1_{X}=0$. Table 1 shows the observed times and event indicators for each of the configurations shown in figure 1 numbered from left to right.

**Table 1.** Observed times $\left\{t_{E},t_{I},t_{R}\right\}$ and event indicators $\left\{1_{E},1_{I},1_{R}\right\}$.

case	observed times	event indicators
1	$\left\{t_{\text{start}},t_{\text{start}},t_{\text{start}}\right\}$	{1, 1, 1}
2	$\left\{t_{\text{start}},t_{\text{start}},T_{R}\right\}$	{1, 1, 1}
3	$\left\{t_{\text{start}},T_{I},T_{R}\right\}$	{1, 1, 1}
4	$\left\{t_{\text{start}},T_{I},t_{\text{stop}}\right\}$	{1, 1, 0}
5	$\left\{T_{E},T_{I},T_{R}\right\}$	{1, 1, 1}
6	$\left\{T_{E},T_{I},t_{\text{stop}}\right\}$	{1, 1, 0}
7	$\left\{T_{E},t_{\text{stop}},t_{\text{stop}}\right\}$	{1, 0, 0}
8	$\left\{t_{\text{stop}},t_{\text{stop}},t_{\text{stop}}\right\}$	{0, 0, 0}
9	$\left\{t_{\text{start}},t_{\text{start}},t_{\text{stop}}\right\}$	{1, 1, 0}
10	$\left\{t_{\text{start}},t_{\text{stop}},t_{\text{stop}}\right\}$	{1, 0, 0}

### 2.6. Dynamical survival analysis likelihood and parameter estimation

For both data sets, we have a sample of size N=5000 histories of infection. To construct a likelihood function, we begin with the density function for the exposure time $T_{E}$. Its density function is the negative derivative of $S_{t}$, so

(2.2) $$f_{T_{E}}(t)=-{\dot{S}}_{t}=\beta I_{t}S_{t}\text{,}$$

where $\beta I_{t}$ can be interpreted as the hazard of infection in a susceptible individual and $S_{t}$ is the corresponding survival function.

For the infection and recovery times ($T_{I}$ and $T_{R}$), we have

(2.3) $$\begin{array}{rl} T_{I}-T_{E} & ∼\text{Exponential}(\delta )\;\text{and}\\ T_{R}-T_{I} & ∼\text{Exponential}(\gamma ), \end{array}$$

and the independence relation

(2.4) $$T_{I}-T_{E}\;⟂\;\;\;⟂\;T_{R}-T_{I}.$$

The probability density for an onset of infectiousness is

(2.5) $$f_{T_{I}-T_{E}}(t)=\delta {\text{e}}^{-\delta t},$$

where δ is the hazard of infectiousness onset among individuals in the E compartment and ${\text{e}}^{-\delta t}$ is the survival function for sojourn time in E. Similarly, the probability density for a recovery is

(2.6) $$f_{T_{R}-T_{I}}(t)=\gamma {\text{e}}^{-\gamma t}\text{,}$$

with constant hazard γ of recovery among those in the I compartment and ${\text{e}}^{-\gamma t}$ is the survival function for sojourn time in I. Table 2 shows the likelihood contribution for each possible configuration shown in figure 1 with the observed times and event indicators from table 1. Note that $1-S_{t_{\text{start}}}=\rho_{E}+\rho_{I}+\rho_{R}$ and that ${\dot{S}}_{t}$ and $S_{t_{\text{stop}}}$ are found by solving the ODEs in equation (2.1), so these are all determined by the parameter vector $\Theta =(\beta ,\delta ,\gamma ,\rho_{E},\rho_{I},\rho_{R})$. We jointly estimated these parameters via maximum likelihood. For numerical stability, we minimized the negative log-likelihood. Because each parameter in Θ has a range on the positive real line, we estimated the natural log of each parameter to improve numerical stability and to prevent point estimates of Θ with negative values (e.g. [28]).

**Table 2.** Likelihood contribution for each case shown in figure 1 and table 1.

case	indicator	likelihood contribution
1	$1_{E}1_{I}1_{R}$	$\rho_{R}$
2	$1_{E}1_{I}1_{R}$	$\rho_{I}\gamma {\text{e}}^{-\gamma (t_{R}-t_{I})}$
3	$1_{E}1_{I}1_{R}$	$(1-S_{t_{\text{start}}})\delta {\text{e}}^{-\delta (t_{I}-t_{E})}\gamma {\text{e}}^{-\gamma (t_{R}-t_{I})}$
4	$1_{E}1_{I}(1-1_{R})$	$(1-S_{t_{\text{start}}})\delta {\text{e}}^{-\delta (t_{I}-t_{E})}{\text{e}}^{-\gamma (t_{R}-t_{I})}$
5	$1_{E}1_{I}1_{R}$	$-{\dot{S}}_{t_{E}}\delta {\text{e}}^{-\delta (t_{I}-t_{E})}\gamma {\text{e}}^{-\gamma (t_{R}-t_{I})}$
6	$1_{E}1_{I}(1-1_{R})$	$-{\dot{S}}_{t_{E}}\delta {\text{e}}^{-\delta (t_{I}-t_{E})}{\text{e}}^{-\gamma (t_{R}-t_{I})}$
7	$1_{E}(1-1_{I})(1-1_{R})$	$-{\dot{S}}_{t_{E}}{\text{e}}^{-\delta (t_{I}-t_{E})}$
8	$\left(1-1_{E})(1-1_{I})(1-1_{R}\right)$	$S_{t_{\text{stop}}}$
9	$1_{E}1_{I}(1-1_{R})$	$\rho_{I}\gamma {\text{e}}^{-\gamma (t_{\text{stop}}-t_{\text{start}})}$
10	$(1-1_{E})1_{I}1_{R}$	$\rho_{E}\delta {\text{e}}^{-\delta (t_{\text{stop}}-t_{\text{start}})}$

To evaluate how well the model estimated parameters at different stages of an epidemic, we repeatedly obtained maximum likelihood estimates using a sliding observation window. We chose an observation window that was 5 days long for our 100-day long simulated epidemic, which died out in roughly 70 days. We moved the observation window in increments of 1 day from days 0 to 36 and recorded the parameter estimates as well as the standard errors. For the Epicast dataset, the epidemic started around day 20 and died out around day 100. We selected a window of 7 days and slid the observation window in units of 1 day from days 25 to 75. We recorded parameter estimates and standard errors for each window.

### 2.7. Prediction

To evaluate our model’s prediction ability with the data generated by a compartmental SEIR model, we first estimated model parameters Θ based on data within an observation window. Then, using the maximum likelihood estimate $\hat{\Theta }$, we estimated S, E, I and R at various times in the future. To generate 95% confidence intervals for these predictions, we generated 1000 samples of Θ from a multi-variate normal distribution with mean $\hat{\Theta }$ and variance equal to the estimated covariance matrix. We simulated the epidemic over the prediction times to get S, E, I and R values for each sampled Θ. Then, we took the 2.5 and 97.5% quantiles of each prediction to get a 95% confidence interval. We used this simulation-based approach to account for nonlinearity in the SEIR model more simply and accurately than an asymptotic method such as the delta method.

For our simulated SEIR data, we were able to make one continuous prediction until the end of the epidemic. For the Epicast data, we made short-term predictions at specific time points because it was not realistic to assume that parameters of the epidemic model used to calculate the likelihood will be constant in time. The Epicast scenario represents a situation more like a real epidemic.

### 2.8. Forecasting the quality of predictions

In the Epicast data and in real epidemic data, we expect the parameters of our approximation to change over time. These changes can stem from systematic differences between the epidemic and the model used in the likelihood as well as from changes in behaviour or policy. Using our local DSA approach to prediction, we designed a diagnostic statistic based on the likelihood function to determine whether a prediction would be poor. We hypothesized that predictions will be poor if parameters are changing rapidly from one window to the next. Note that the converse—that no change in parameters implies a good prediction—is not necessarily true.

Figure 2 shows a schematic of our diagnostic procedure. In the likelihood ratio test (LRT), the likelihood under the null model uses estimated parameters from window A, and the likelihood under the alternative model uses estimated parameters from window B. The LRT statistic serves as a metric for whether there has been a change in parameters from one window to the next. Then, we make predictions at times $t_{C}$ and $t_{D}$, based on parameter estimates from the combined window A + B. The measure of the prediction quality is the ratio of the predicted and empirical changes in the cumulative hazard of infection $-\ln S_{t}$. For the prediction at time $t_{C}$, the change in the cumulative hazard of infection is $\Delta H=\ln S_{t_{B}}-\ln S_{t_{C}}$, where $t_{B}$ is the endpoint of window B. For the prediction at time $t_{D}$, $\Delta H=\ln S_{t_{B}}-\ln S_{t_{D}}$. The predicted change in the cumulative hazard, $\Delta H_{\text{pred}}$, is based on ${\hat{S}}_{t}$ calculated using the maximum likelihood estimate $\hat{\Theta }$. The empirical change, $\Delta H_{\text{emp}}$, is based on the true $S_{t}$, which is either that of the true SEIR model or that of the population in Epicast. To map the ratio of cumulative hazards onto the entire real line, we used a log transformation. To equally penalize predictions that were too low and too high, we took the absolute value of the log. Thus, the diagnostic statistic is

(2.7) $$\begin{array}{r} \left|\log\left(\frac{\Delta H_{\text{pred}}}{\Delta H_{\text{emp}}}\right)\right|. \end{array}$$

To evaluate the ability of the LRT within the window A + B to indicate poor predictions at times $t_{C}$ and $t_{D}$, we plotted this statistic against the log of the likelihood ratio statistic. Details about the implementation can be viewed in the R code, available in our Zenodo repository [29].

**Figure 2.**
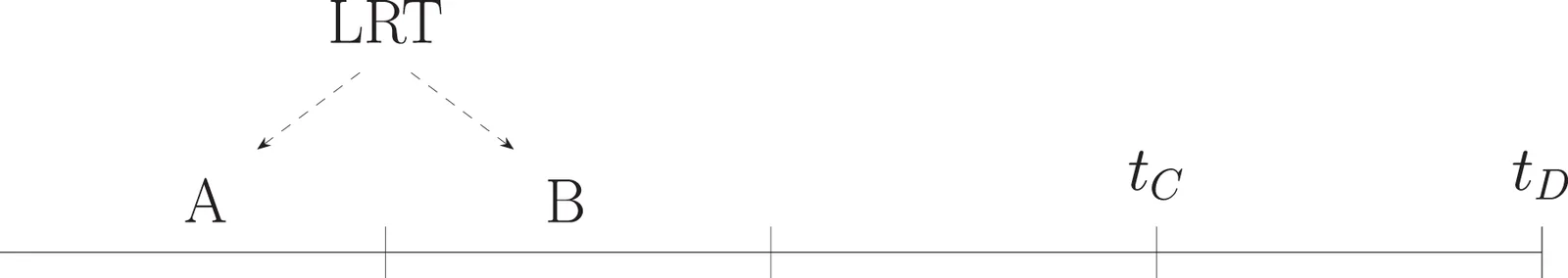
Schematic of LRT diagnostic test. Predictions are made based on fitted window (A + B).

## 3. Results

### 3.1. Local parameter estimation in simulated SEIR data

Using our simulated data, we generated 5000 human sensors using the process described in §2.3 and estimated parameters over a sliding window at each time point. Figure 3a shows point and interval estimates of each parameter (on the log scale used for estimation) at each window start time. It also shows point and interval estimates of $\ln R_{0}=\ln\beta -\ln\gamma$. Figure 3b shows the corresponding standard errors. Figure 4 shows the corresponding point and interval estimates of each parameter on the most natural scale for interpretation of the epidemic model. The estimates of β, δ, γ and $R_{0}$ remained consistent throughout the epidemic. When the underlying epidemic matches the model used in the DSA likelihood up to unknown parameter values, parameter estimation does not depend on the location of the observation window. The standard errors reach minimums near the peak of the epidemic (≈20 days for E and ≈30 days for I), which indicates that information about the epidemic is concentrated near the peak. However, accurate and precise estimates can be obtained at any point in the epidemic.

**Figure 3.**
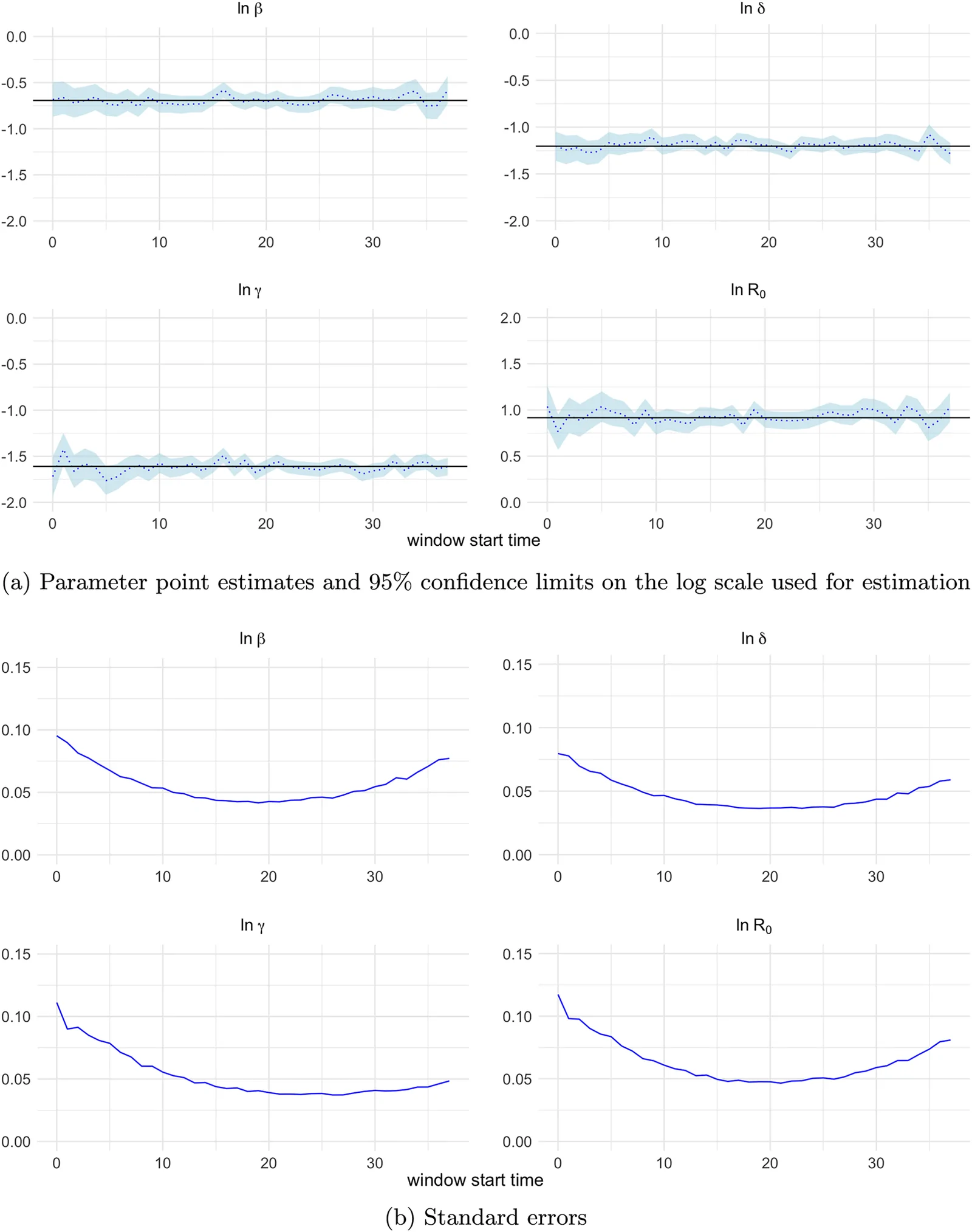
Parameter estimates as the 5-day observation window moves across time in the simulated SEIR data. In panel (a), each dot represents an estimated parameter on the log scale used for estimation; 95% confidence intervals shaded in blue and black lines show the true values. Panel (b) shows the corresponding standard errors. $R_{0}=\beta /\gamma$ is the basic reproduction number.

**Figure 4.**
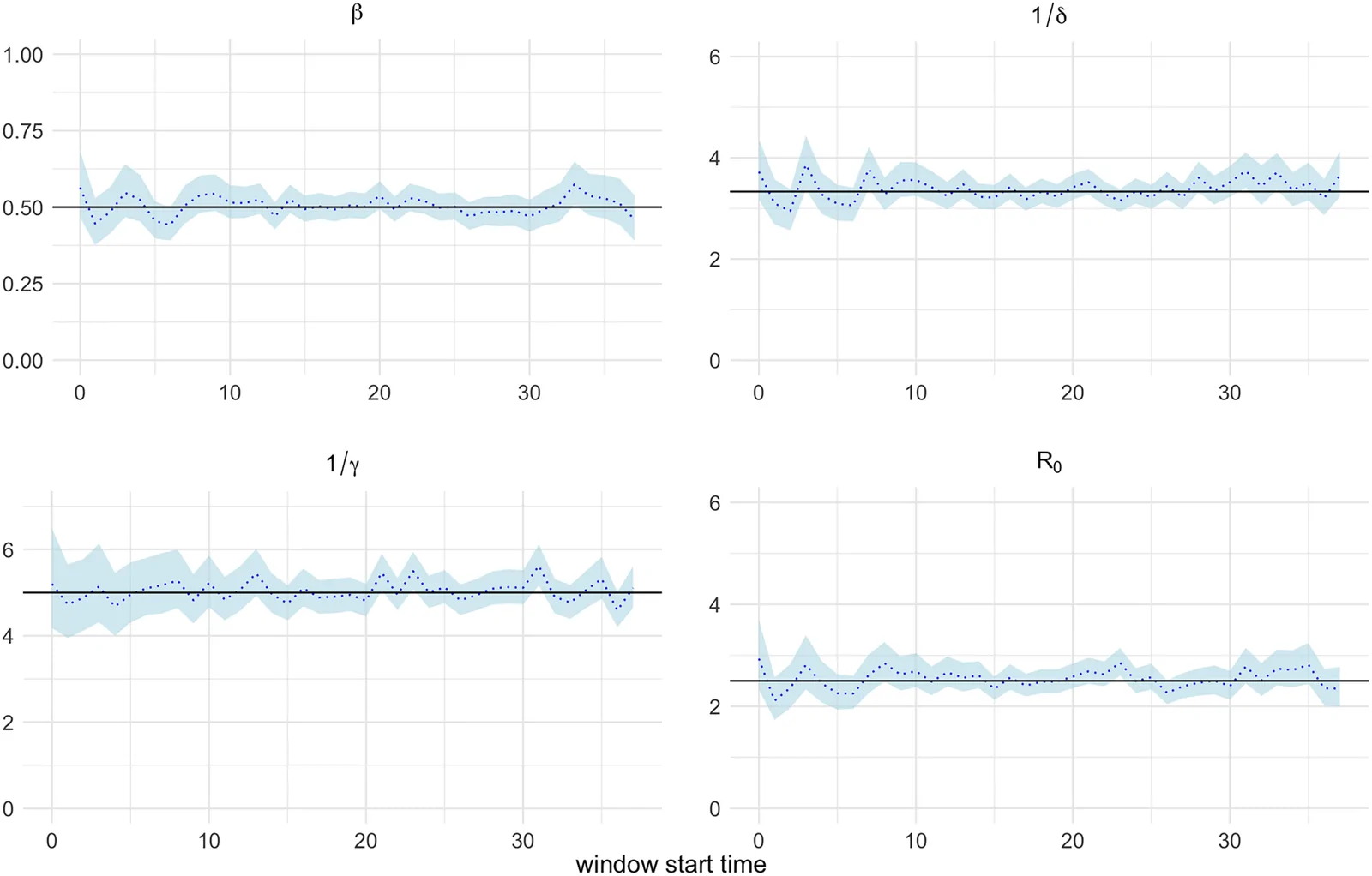
Point and interval estimates of β, 1/δ, 1/γ and $R_{0}$ corresponding to the point and interval estimates shown in figure 3a. Each parameter is shown on the scale most relevant for interpretation of the epidemic model. Note that 1/δ is the mean latent time and 1/γ is the mean infectious period.

### 3.2. Prediction in simulated SEIR data

Here, we evaluate the model’s ability to predict the decay of susceptibles and the prevalence of infected individuals—that is, the S and I curves. Figure 5 shows a sample prediction through the end of the epidemic based on a 5-day observation window. The solid lines represent the true epidemic curves, while the dashed lines show the predicted epidemic curves and the dotted lines show the associated 95% confidence intervals. The observation window is shaded in grey. Even with a narrow observation window early in the epidemic, prediction of the S and I curves is accurate all the way to the end. This simulation occurred under ideal conditions, so we do not anticipate similar performance in practice.

**Figure 5.**
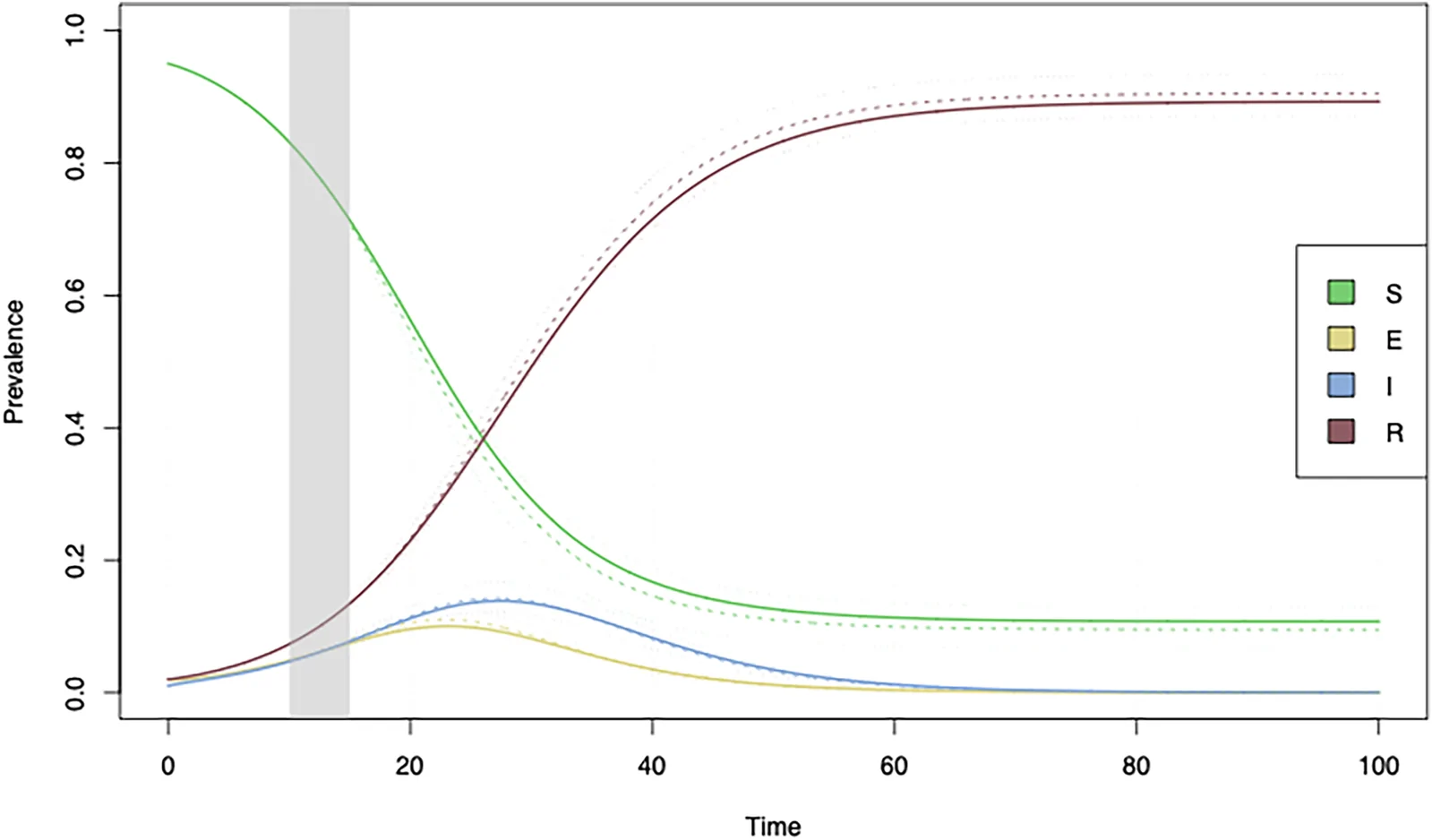
Predicted S, E, I and R (dashed lines) based on the shaded grey area, which is a 5-day observation window on days 10–15. Estimated 95% confidence limits are shown with dotted lines. Predictions based on 5000 human sensors are reliable throughout the epidemic.

To test prediction more thoroughly, we conducted a simulation study of the procedure illustrated in figure 5. We repeated it 500 times with various start times for the observation window: 5, 15, 25 and 35. For each of these start times, we calculated coverage probabilities for the predicted S and I curves at various time points after the end of the observation window. The results are summarized in table 3, which shows that coverage probabilities are consistently close to 95% for S and often higher for I. When the data generation process is correctly specified in the model, we get reliable predictions of S and I from an observation window anywhere in the epidemic.

**Table 3.** Coverage probabilities for predicted S and I curves over 500 runs using a window size of 5, for various prediction times and starting points $t_{\text{start}}$.

$(t_{\text{start}},t_{\text{stop}}$)	prediction time	coverage prob. (S)	coverage prob. (I)
(5,10]	15	0.934	0.964
	25	0.944	0.970
	30	0.946	0.972
	40	0.950	0.974
	50	0.956	0.978
	60	0.958	0.978
(15,20]	25	0.944	0.978
	30	0.950	0.982
	40	0.954	0.978
	50	0.958	0.978
	60	0.952	0.976
(25,30]	35	0.956	0.980
	40	0.944	0.966
	50	0.944	0.968
	60	0.946	0.968
(35,40]	45	0.948	0.976
	50	0.944	0.978
	60	0.944	0.974

### 3.3. Local parameter estimation in Epicast data

Using data generated from Epicast, we repeated the parameter estimation with a sliding window from §3.1. Although these data also come from a simulation, Epicast has a much higher degree of complexity and is meant to imitate real-world data. With the Epicast data, as in the real world, our estimates are not based on knowledge about the structure or parameters of the underlying epidemic. We used a 7-day window to account for weekly effects owing to work and school attendance patterns for agents in Epicast.

Figure 6a shows point and interval estimates of each parameter (on the log scale used for estimation) at each window start time. It also shows point and interval estimates of $\ln R_{0}$. Figure 6b shows the corresponding standard errors. Figure 7 shows corresponding point and interval estimates of each parameter on the most natural scale for interpretation of the epidemic model. These are analogous to figures 3a,b and 4, respectively. The parameter estimates for the Epicast data are much less stable and there is clear evidence of time-varying parameters, indicating that these data truly do not fit a standard SEIR model structure. This is not surprising given the complex individual agent behaviour programmed into the Epicast model.

**Figure 6.**
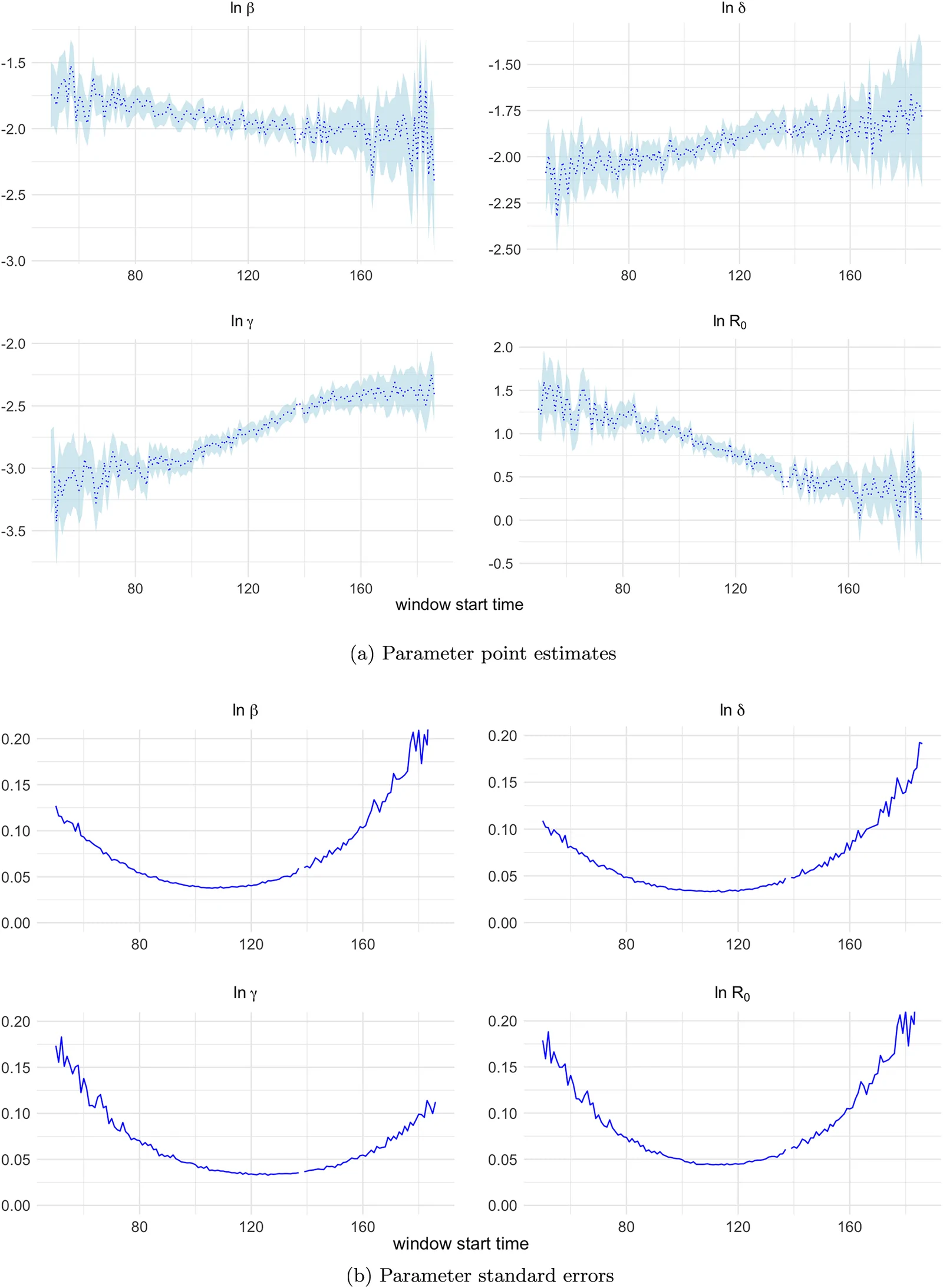
Parameter estimates as the 7-day observation window moves across time in the Epicast data. In panel (a), each dot represents an estimated parameter on the log scale used for estimation and 95% confidence intervals are shaded in blue. Panel (b) shows the corresponding standard errors.

**Figure 7.**
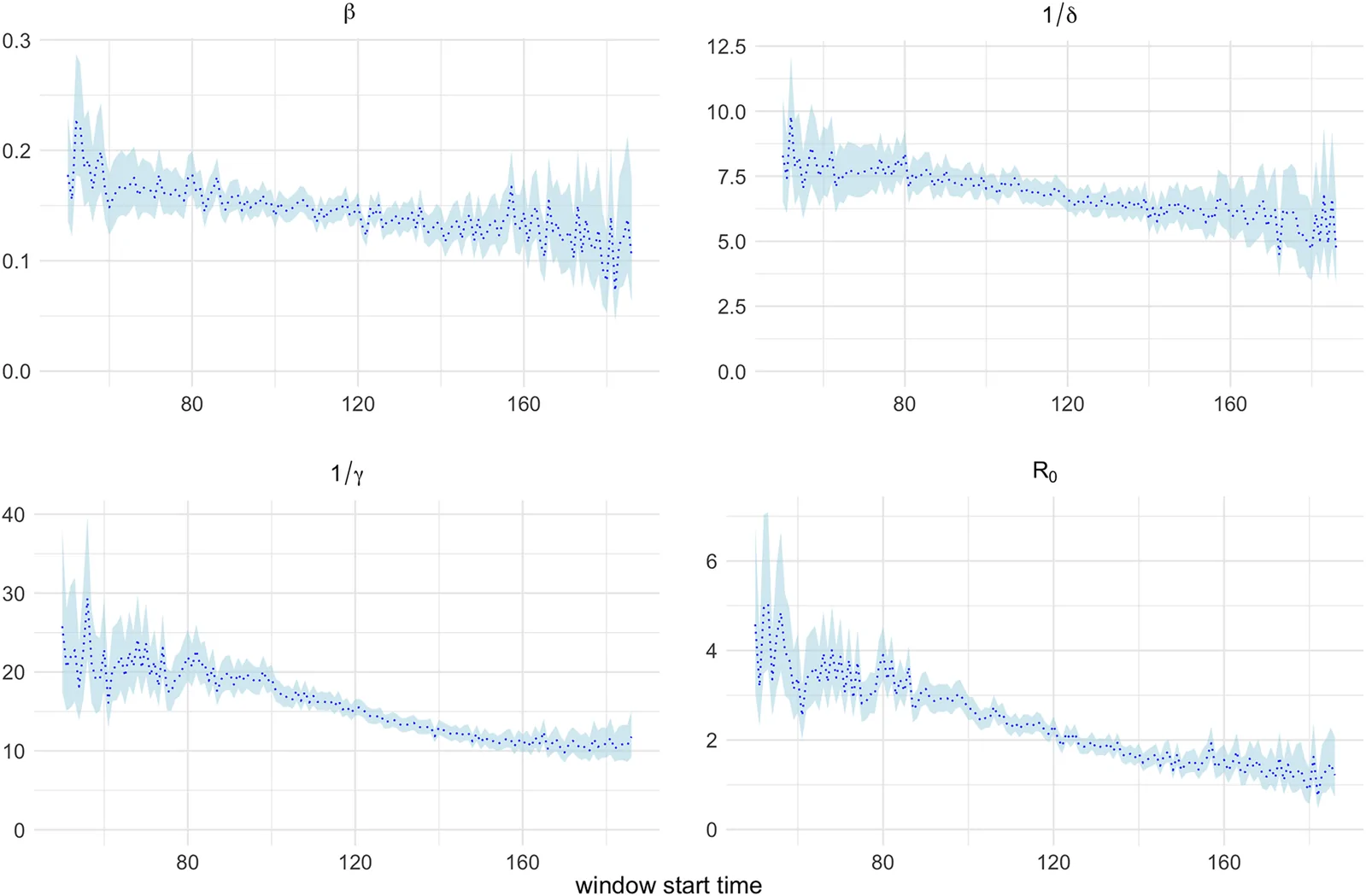
Point and interval estimates of β, 1/δ, 1/γ and $R_{0}$ corresponding to the point and interval estimates shown in figure 6a. Each parameter is shown on the scale most relevant for interpretation of the epidemic model. Note that 1/δ is the mean latent period and 1/γ is the mean infectious period.

### 3.4. Local prediction in Epicast data

Because of systematic variation in parameter values, we cannot expect DSA to make good long-term predictions for the Epicast data. Instead, we propose using short windows of fitted data to predict short windows into the future. To implement local prediction, we fed 7-day windows of data into our model and estimated parameters for each window by maximum likelihood. Figure 8 shows fitted segments plotted on top of the empirical data. Based on the estimated parameters in each 7-day window, we made predictions for the next 7-day window. Figure 9 shows predicted segments plotted on top of the empirical data. In this piecewise manner, we traversed the entire timeline.

**Figure 8.**
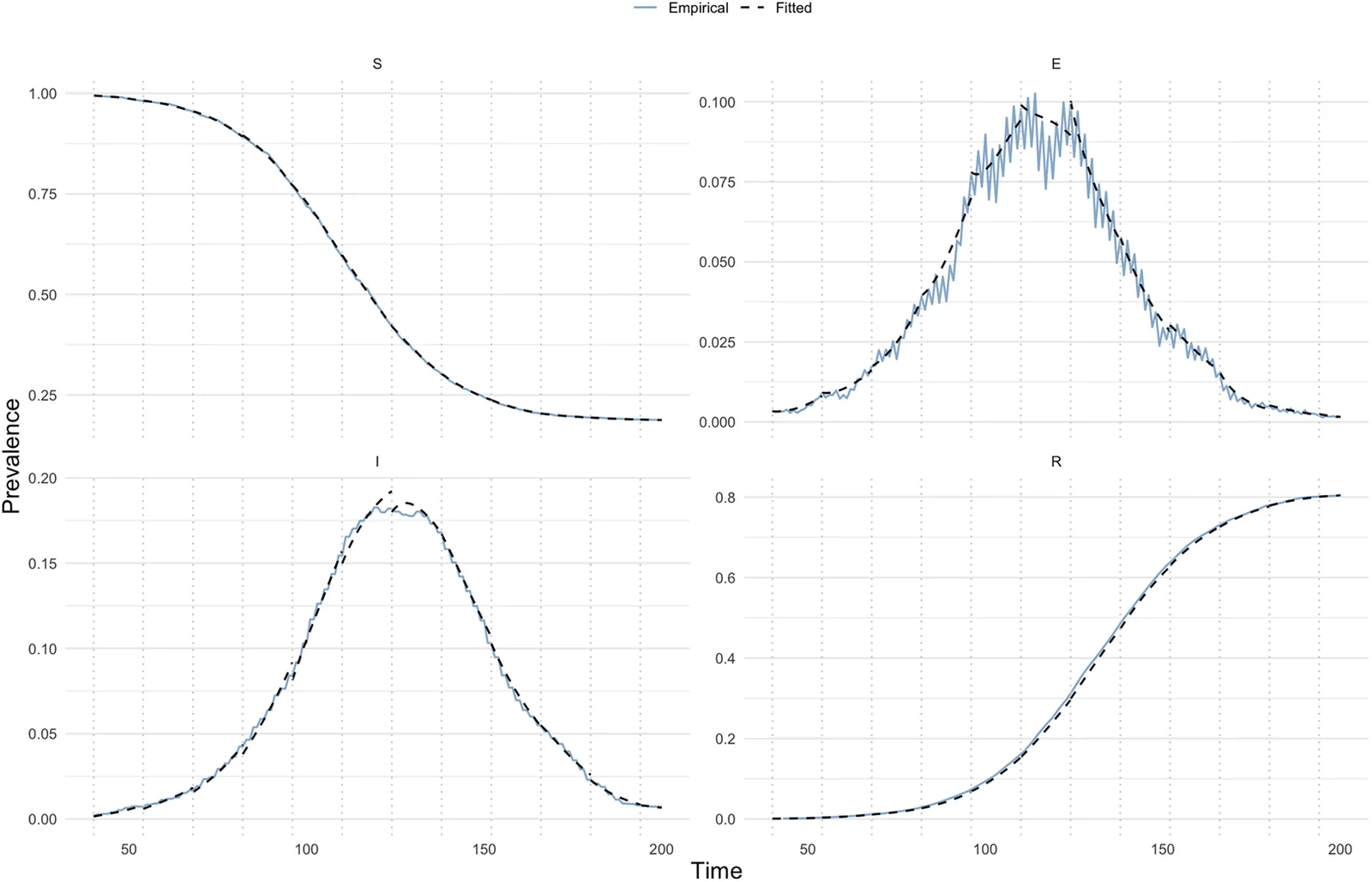
Panels show epidemic curves S,E,I and R. Dotted grey lines divide the epidemic into 7-day observation windows. Blue curves are empirical data from Epicast, and dashed black curves are **fits** based on a DSA model fit to data from each window.

**Figure 9.**
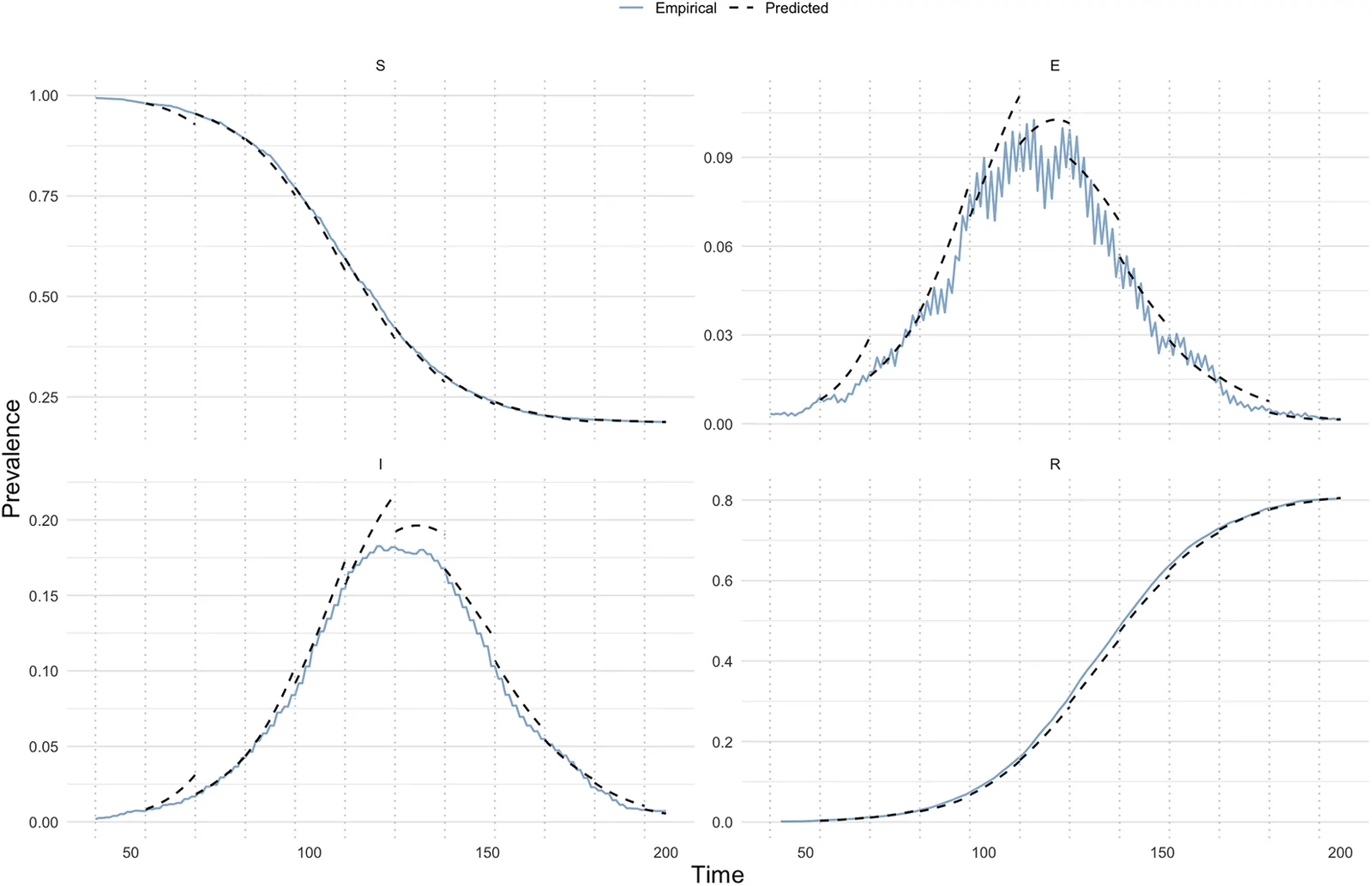
Epidemic curves S, E, I and R. Dotted grey lines divide the epidemic into 7-day observation windows. Blue curves are empirical data from Epicast, and dashed black curves are **predictions** based on the DSA fit to observations from the previous 7-day window.

In the S and R curves, the predictions are very close throughout, making it hard to distinguish between the empirical and the predicted curves. However, there are visible instances of over- or under-estimation in predictions in the E and I curves. Within each interval (separated by dotted grey lines), the predictions slowly drift farther from the empirical data. However, they promptly return to the empirical curves at the start of the next interval, highlighting the benefits of using a local approach.

### 3.5. Forecasting the quality of predictions in the Epicast data

To evaluate whether we can forecast the quality of predictions in the Epicast data, we adapted the process illustrated in figure 2. We calculated a LRT over two weeks of observation (A and B) and made predictions one week ($t_{C}$) and two weeks ($t_{D}$) after the end of observation. We randomly sampled 5000 agents from Epicast and computed the change in the ratio of the cumulative hazards and the LRT statistic at each 7-day interval. We repeated this procedure 100 times with a new sample each time. Figure 10 shows predictions at one week, and figure 11 shows predictions at two weeks. The y-axis of each plot contains information on the prediction quality, with zero corresponding to a perfect prediction and larger values indicating more bias. As the LRT statistic values increase (i.e. parameters are less stable over time), there is a clear increase in prediction bias. The black vertical line represents the critical value for a LRT with a 5% significance level. To the right of this line, there is a strong correlation between the LRT statistic and the quality of predictions, supporting the hypothesis that a change in parameters between intervals A and B is associated with poor predictions. To the left of this line, there is a much weaker relationship. When parameters are stable within the observation window, we may get good predictions (if the best-fitting parameter values stay constant) or bad predictions (if the best-fitting parameter values start to change). Note that the 5% significance level used here simply gives us a convenient way to draw a boundary between cases where DSA model parameters are or are not stable over time.

**Figure 10.**
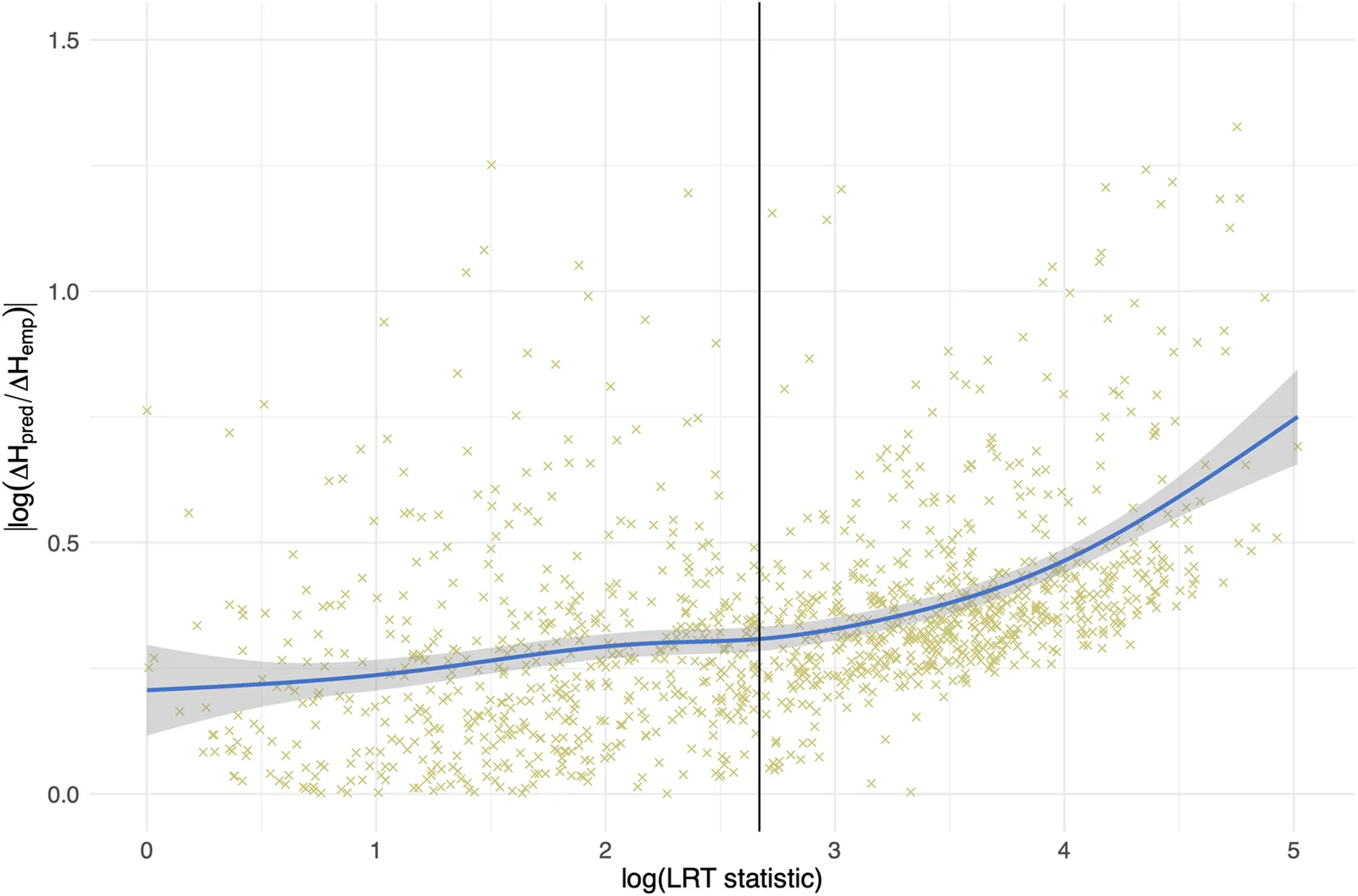
Log of the LRT statistic and the absolute value of $\log(\Delta H_{\text{pred}}/\Delta H_{\text{emp}})$ for 100 iterations of an Epicast agent network, using predictions at one week after the end of the observation window. The critical value for a 5% significance level is shown with a black vertical line, and a local regression line with pointwise 95% confidence limits is plotted in blue.

**Figure 11.**
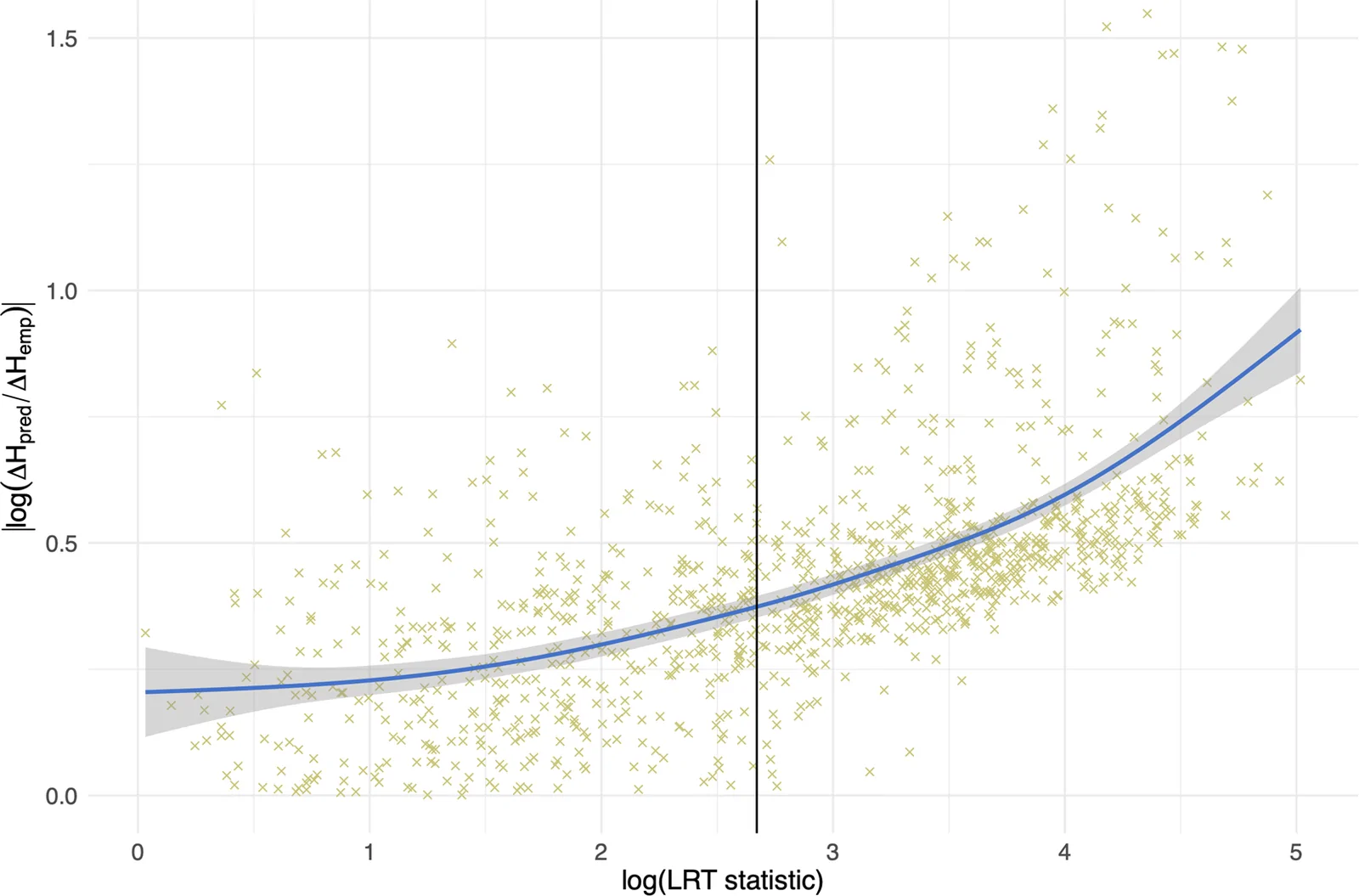
Log of the LRT statistic and the absolute value of $\log(\Delta H_{\text{pred}}/\Delta H_{\text{emp}})$ for 100 iterations of an Epicast agent network, using predictions at two weeks after the end of the observation window. The critical value for a 5% significance level is shown with a black vertical line, and a local regression line with pointwise 95% confidence limits is plotted in blue.

## 4. Discussion

This study strongly suggests that human sensor networks and local DSA can be used to improve tracking and prediction of epidemics. Human sensor networks, where individuals are sampled from the population and followed longitudinally, allow greater control over bias than approaches based on time-series data from clinical testing. The local DSA approach allows these data to be analysed using likelihood-based inference and tools from survival analysis, which allows more flexibility and more accurate uncertainty quantification than current methods. Here, we have fitted mass action models, but the DSA approach can also be used to fit models based on configuration model networks [30] which can allow for more realism within a simple model.

When the underlying epidemic data generating process is known (up to parameter values), the model produces reliable parameter estimates and predictions based on almost any observation window in the past. When the underlying epidemic is unknown, local DSA can capture complex dynamics by allowing time-varying parameters in the epidemic model used to calculate the likelihood. This is especially important in the case of novel outbreaks where there are little to no empirical data to guide modelling efforts. The COVID-19 Infection Survey implemented by the Office for National Statistics in the UK is similar in many ways to a human sensor network, and it generated valuable insights into COVID-19 transmission [31,32]. However, the human sensor networks we consider here would generally have a higher frequency of testing and a smaller number of households.

It is remarkable that this approach gives us some ability to forecast poor predictions of epidemic spread. When parameters are changing during observation, we are likely to get poor predictions. When parameters are not changing during observation, we may or may not get a good prediction. It is possible that comparing changes in parameters over time in different models could allow us to choose the DSA model most likely to produce good predictions.

With data from a human sensor network, survival analysis can be used to track the spread of infection without any epidemic modelling. Because we can handle right censoring and left truncation (i.e. delayed entry), it is possible to account for drop out of current participants and recruitment of new participants. This can be used to limit the burden on individual participants. Although we used only a random sample from the population here, the human sensor network design could be extended using all of the tools from survey sampling to obtain more accurate estimates of spread in specific subpopulations. This could be important in cases where certain subpopulations are at higher or lower risk of infection than others.

One limitation of this work is that we are assuming perfect knowledge of event times for each individual. In a real human sensor network, these event times would often be interval censored. Additionally, we would be unlikely to observe exposure times and infection times separately. However, in a study using data from an Ebola outbreak, Vossler *et al.* [33] were able to estimate epidemic parameters using only approximate infection and recovery times with DSA, which indicates that the method can be adapted to real data. Another limitation is that the decision for the size of the observation window is largely arbitrary. For the Epicast data, we chose a 7-day window based on prior knowledge about human behaviour and some knowledge about the model regarding agent interaction in various settings (e.g. workplace and school). For other epidemic data, there could be several factors that contribute to an ideal window size. Making the window too large risks generating poor predictions owing to bias, but making the window too small risks overfitting the data and generating poor predictions owing to high variance. This is the same bias–variance trade-off faced by time-series methods [15], and further research could explore a systematic way to choose a window size or to define an adaptive window size.

Accurate surveillance is critical for epidemic control, and accurate short-term forecasts could be extremely useful. Based on the results presented above, we believe that learning to handle the logistical challenges of designing and implementing a human sensor network is an important area for future research. Communities can be better prepared to face future epidemics if the infrastructure needed to recruit, follow and analyse human sensor networks is in place ahead of time. When prevalence and incidence are very low in the early stages of an epidemic, we believe that time-series methods such as those outlined by Gostic *et al.* [15] have an important role to play in surveillance. The transition between time-series and human sensor networks—or how these surveillance systems can complement each other systematically—is another important area for future research.

## Data Availability

All data and code that support the findings of this study are available on Zenodo: http://zenodo.org/records/17604801.
